# Capacity utilization and the cost of primary care visits: Implications for the costs of scaling up health interventions

**DOI:** 10.1186/1478-7547-6-22

**Published:** 2008-11-13

**Authors:** Taghreed Adam, Steeve Ebener, Benjamin Johns, David B Evans

**Affiliations:** 1 Alliance for Health Policy and Systems Research, World Health Organization, Geneva, Switzerland; 2 Knowledge Management and Sharing, World Health Organization, Geneva, Switzerland; 3 Johns Hopkins University, Baltimore, USA; 4 Health Systems Financing, World Health Organization, Geneva, Switzerland

## Abstract

**Objective:**

A great deal of international attention has been focussed recently on how much additional funding is required to scale up health interventions to meet global targets such as the Millennium Development Goals (MDGs). Most of the cost estimates that have been made in response have assumed that unit costs of delivering services will not change as coverage increases or as more and more interventions are delivered together. This is most unlikely. The main objective of this paper is to measure the impact of patient load on the cost per visit at primary health care facilities and the extent to which this would influence estimates of the costs and financial requirements to scale up interventions.

**Methods:**

Multivariate regression analysis was used to explore the determinants of variability in unit costs using data for 44 countries with a total of 984 observations.

**Findings:**

Controlling for other possible determinants, we find that the cost of an outpatient visit is very sensitive to the number of patients seen by providers each day at primary care facilities. Each 1% increase in patient through-put results, on average, in a 27% reduction in the cost per visit (p < 0.0001), which can lead to a difference of up to $30 in the observed costs of an outpatient visit at primary facilities in the same setting, other factors held constant.

**Conclusion:**

Variability in capacity utilization, therefore, need to be taken into account in cost estimates, and the paper develops a method by which this can be done.

## Background

Making the best use of available resources is vital in developing countries that are struggling to improve public health with limited funds. This has become even more urgent following their ambitious commitment to achieve the Millenium Development Goals (MDGs) and the realization that funding is not yet sufficient to allow interventions to be scaled up sufficiently to do so [1]. Consequently, demand for information on how much additional funding would be required to attain the MDGs has increased, and in response, a number of studies have tried to estimate the costs countries are likely to face in further scaling-up health interventions. Most current estimates are likely to be substantially incorrect, however, with perhaps the most important problem the assumption that the unit costs of delivering services – for example, the costs per visit to a primary health facility, or the costs of a day in hospital – will not change as coverage increases or as more interventions are delivered together [2,3]. This is most unlikely [4,5].

Increased utilization due to scaling up may have a positive or negative impact on unit costs, depending on the current level of capacity utilization at primary facilities. For example, in facilities functioning at less than full capacity, unit costs are likely to fall in the short term with increases in output, as more services are delivered by existing facilities – fixed costs are distributed over a larger number of recipients. But in the longer run, unit costs could rise if new facilities have to be built in sparsely populated areas or it becomes increasingly difficult to attract the remaining people in need to seek care. The likely existence of these "economies" and "diseconomies of scale" means that information on the current and expected levels of capacity utilization at different stages of scaling up is key to identifying the true costs of expanding population coverage. This information is rarely reported or collected, however, and even if it is available, there are no guidelines on how to take them into account when estimating unit costs at primary facilities [2,6].

Another limitation of current analyses is that the cost of an outpatient visit or inpatient day used to estimate overall costs are usually derived from a small number of health facilities or programs, sometimes only one [7,8]. This is likely to be misleading given the large variability in capacity utilization across facilities within the same country – by chance the studied facilities or programs might have higher, or lower, levels of capacity utilization than other facilities or programs, leading to an under- or over-estimate of national costs [9,10].

While this is an indisputable theoretical possibility, the question remains whether it will be important in practice. The main objective of this paper is to measure the impact of the level of capacity utilization, in this case patient load, on the cost of a visit to a primary health care facility. The paper will examine the extent of the variation in this cost due to variations in capacity utilization, and will derive a method that can be used to adjust unit costs for different levels of capacity use. This work is part of WHO-CHOICE project with the overall objective to estimate the costs and health impact of a large number of health interventions at different levels of efficiency and population coverage levels. For more detail about WHO-CHOICE methods and results see .

## Methods

### Data

Part of the unit cost data was obtained from a number of WHO-commissioned studies in a representative sample of facilities in countries where these data were particularly scarce, see Appendix 1 for the list of countries. In addition, data were extracted from manuscripts published in the available indexed search engines: Medline, Econlit, Social Science Citation Index, regional Index Medicus, Eldis (for developing-country data), Commonwealth Agricultural Bureau (CAB), and the British Library for Development Studies Databases [7,10-22].

**Appendix 1:** Countries included in the analysis.

**Country**	**N**	**Country**	**N**
Australia	16	Mongolia*	13
Benin*	39	Morocco*	17
Brazil*	69	Nepal	2
Cameroon*	15	New Zealand	2
Canada	17	Norway	2
China*	40	Pakistan*	145
China (Jiangsu)*	13	Peru	1
China (Shanghai)*	46	Poland	2
Colombia	1	Republic of Korea	11
Ecuador*	67	Russian Federation*	32
Egypt*	35	Sierra Leone	1
Finland	1	Sri Lanka	6
Gambia	2	Sudan*	9
Ghana	5	Sweden	5
India	1	Syrian Arab Republic*	8
Jordan*	12	Thailand*	96
Kenya*	73	Tunisia*	18
Kuwait*	24	Turkey	5
Lebanon*	10	Uganda	5
Lesotho	1	United Arab Emirates*	13
Luxembourg	4	United Kingdom	5
Malawi	5	United Republic of Tanzania*	87
Mexico	2	Viet Nam*	1

**Total**	**984**		

N = number of health facilities per country for which annual unit costs were obtained and included in the analysis.

*unit cost data at least partly collected from commissioned studies

The search terms used were: "costs and cost analysis" and health centre or the abbreviations HC (health centre) or PHC (primary health centre) or outpatient care. The language sources searched were English, French, Spanish and Arabic; no Arabic study was found. Additional data were also obtained from a number of studies in the grey literature, from such sources as electronic databases, government regulatory bodies, research institutions, and individual health economists known to the authors [7,8,11-17,19,23-52].

Data from all sources were entered in a standard data-extraction template, including all variables that may contribute to understanding the relationship between unit costs and their determinants. The cost per outpatient visit at primary care facilities was the dependent variable and the possible explanatory variables included: ownership; total number of outpatient visits; types of costs included in the original cost study (e.g., capital, drugs, laboratory and diagnostics); whether reported costs were based on costs or charges; the total number of full time equivalent health care providers at the facility; the reference year for cost data; the currency; and the methods the costing studies had used to allocate joint costs. Data on the number of outpatient visits and the number of providers were used to calculate the indicator of capacity utilisation – the average number of visits per provider per day, if this was not readily reported in data sources. The number of providers was the full time equivalent number of staff, regardless of skill, who examined or treated patients. Data were available for 44 countries with a total of 984 observations. See Appendix 1 for the list of countries.

In addition, information on aggregate variables reflecting socio-economic or other characteristics that may explain part of the variability in unit costs was also collected. The variables included *GDP per capita *[53], which has been used as a proxy for the level of technology [9,10,54-56], labour productivity [57], and the overall level of demand for health care in different studies [58]. *Population density *[59], which controls for access-related efficiency gains or losses due to geographical and demographic characteristics of various settings was also included. Finally, dummy variables indicating whether a country was an *oil producer *(i.e. OPEC member) or if the country had a c*ommunist regime *either now or in the recent past, were also used. In the former case, it might be that costs are higher than would be expected from the level of GDP per capita alone because of inflows of foreign exchange and foreign workers. In the latter, cost levels might be lower than expected due to the historical ability of these countries to control prices and wages.

Prior to the analysis, consistency checks were performed and questionable data queried with the study authors, or omitted if explanations could not be found.

Finally, costs were converted to 2000 US dollars using GDP deflators and official exchange rates [60]. STATA software was used for analysis [61].

### Data imputation

Before model selection, potential variables for inclusion in the analysis were explored for missing data. Only two variables were concerned, the number of visits per provider per day and the total number of annual visits, where data was missing in 70% and 18% of the observations, respectively. Although the percent of missing data in the former was relatively high, we decided that the bias introduced by restricting the analysis to those observations with complete data would be larger than that caused by imputing missing data combined with appropriate uncertainty analysis [62]. A requirement for using imputation methods is that data are missing at random, which we believe is the case here, since the main reason data are not reported is that it is not yet standard practice in the costing literature.

Multiple imputation techniques are the most suitable for our case, where the observed values for other settings, as well as relevant covariates, are used to predict a distribution of likely values for the unobserved data. It also allows subsequent analysis to take account of the level of uncertainty surrounding each imputed value [63-66]. The statistical model used for multiple imputation is the joint multivariate normal distribution, using *Amelia software *[64,67-69]. One of its main advantages is that it produces reliable estimates of standard errors, and through the introduction of random error into the imputation process, it considerably reduces potential biases in the imputed data [63]. Detail of the estimation process and handling of the model output can be found elsewhere [10].

### Model specification

Empirical cost function studies – i.e. studies that relate unit costs to the level of output – have been mainly interested in estimating hospital costs. None to our knowledge have focused on primary care facilities. We followed the basic approach used to estimate hospital cost functions by Lombard et al (1991) and Adam et al (2003) and (2006) [9,10,70]. The relationship between the cost per visit and the level of capacity use, as well as other possible determinants, was explored using multiple regression analysis – Ordinary Least Squares (OLS) was used. The dependent variable and all continuous explanatory variables explored in this model were transformed into natural logarithms, as this specification resulted in a residual plot that best approximated a normal distribution – a requirement of OLS regressions. Natural logs have the added advantage that coefficients can be readily interpreted as elasticities, offering a straightforward measure of the impact of capacity utilization on costs, the main focus of this analysis [71,72]. In addition, robust estimation methods were used, using the "robust" command in STATA [61], to control for clustering resulting from the inclusion of multiple observations per country in the study [73].

The functional specification of the OLS regression model may be written as:

(1) $$\begin{array}{cc} \ln UC_{i}=\alpha_{0}+\sum_{i=1}^{n}\alpha_{i}{\text{X}}_{i}+e_{i}, & i=1\ldots n \end{array}$$

where ln *UC*_*i *_is the natural log (ln) of cost per outpatient visit in 2000 US $ in the *ith *facility; *α*_0 _and *α*_1...*n *_are the estimated parameters; the *X*_*i *_are the explanatory variables described earlier, transformed into natural logarithms for continuous variables [60]; and *e *denotes the error term.

The cost of an outpatient visit is expected to be positively correlated with GDP per capita; the inclusion of capital, ancillary (laboratory and other diagnostic tests) or drug costs in the original costing; and whether the country produced oil. We expected costs to be negatively correlated with the number of visits per provider per day, our variable of interest, and population density; and lower in public compared to private facilities and in countries that had been under communist regimes.

Interaction terms were also tested, such as the interaction between capacity utilization and GDP per capita. Only variables that were consistently significant in the different models were included in the final model that was selected based on econometric grounds.

Finally, to estimate the value of the unit cost per outpatient visit that would be expected for given values of the independent variables, the estimated dependent variable was re-transformed from logarithms to natural units using the Duan smearing factor [74]. The Duan smearing factor is used because one of the implicit assumptions of using log-transformed models is that the least-squares regression residuals in the transformed space are normally distributed. In this case, back-transforming to estimate unit costs gives the median and not the mean. The smearing method described by Duan (1983) corrects for the back transformation bias [74]. This was done by multiplying the anti log of the product of the model by 1.45, the smearing correction factor derived from our model.

### Model-fit

Various regression diagnostics were used to judge the goodness-of-fit of the model. They included residual plots of the residual versus fitted values, "hettest" to test heteroskedasticity of the model variables, the variance inflation factors to test for multicollinearity, and estimates of adjusted R square and F statistics of the regression model [61].

### Sensitivity analysis

Sensitivity of the results to imputation of missing data was explored by running the models with and without imputation.

## Results

Table 1 shows the variable names, description and results of the model with the best statistical fit. The adjusted R-square of the combined regressions from the five imputed datasets is 0.52, with an F statistic of 258 (p < 0.0001). All other regression diagnostic showed a good fit; the variance inflation factors ranged between 1.27 and 1.30 (VIF more than 20 indicates multicollinearity) [61] and the residual plots had a mean of zero with no specific pattern of distribution.

**Table 1:** Ordinary Least square regression results, using robust estimation methods, N = 984

Variable	Description	β Coef	SE	T	P
Ln GDP per capita	Natural log of GDP per capita in 2000 US $	0.6219	0.030	21.08	<0.001
Ln visits per provider per day	Natural log of number of visits per provider per day	-0.2756	0.039	-7.16	<0.001
Capital costs	Dummy variable for inclusion of capital costs. Included = 1	0.7759	0.073	10.70	<0.001
Communist	Dummy for communist and Ex communist	-0.466	0.109	-4.26	<0.001
Public	Dummy for public facility. Public = 1	-0.2541	0.109	-2.34	0.019
Constant		-2.9060	0.285	-10.19	<0.001

Dependent variable: Natural log cost per outpatient visit in 2000 US $

Adjusted R^2^= 0.52

F statistic = 258

p of F statistic < 0.00001

The signs of the coefficients are consistent with our hypotheses; the cost per visit is positively correlated with GDP per capita and the inclusion of capital costs, [10] while the number of visits per provider per day; communist or ex-communist countries; and public as opposed to private ownership of facilities, are associated with a lower cost per visit. The other independent variables did not have a statistically significant impact on costs for our data set. The elasticity of cost per visit to changes in GDP per capita, while positive, is less than one (<0.0001). This means that while outpatient costs per visit are higher in countries with higher levels of GDP per capita, they increase at a slower rate than the rise in GDP. This is consistent with previous findings of the relationship between unit cost of hospital care and GDP per capita [10].

In terms of capacity utilization, the results show that each 1% increase in the number of patients seen per provider per day is associated with 27% reduction in the cost per visit, everything else kept constant (<0.0001).

The sensitivity of the results to the imputation of missing data was explored. The signs and order of magnitude of the coefficients were stable with or without amputation, see Table 2.

**Table 2:** Ordinary Least square regression results, using robust estimation methods – model without imputation of missing data, N = 250

Variable	Description	β Coef	SE	T	P
Ln GDP per capita	Natural log of GDP per capita in 2000 US $	0. 847	0.031	27.12	<0.001
Ln visits per provider per day	Natural log of number of visits per provider per day	-0.32	0.06	-5.33	<0.001
Capital costs	Dummy variable for inclusion of capital costs. Included = 1	0.14	0.10	1.34	0.182
Communist	Dummy for communist and Ex communist	-1.16	0.17	-6.64	<0.001
Public	Dummy for public facility. Public = 1	-0.39	0.24	-1.59	0.114
Constant		-4.15	0.33	-12.50	<0.001

Dependent variable: Natural log cost per outpatient visit in 2000 US $

Adjusted R^2^= 0.658

F statistic = 152.40

p of F statistic < 0.00001

**Figure 1:**
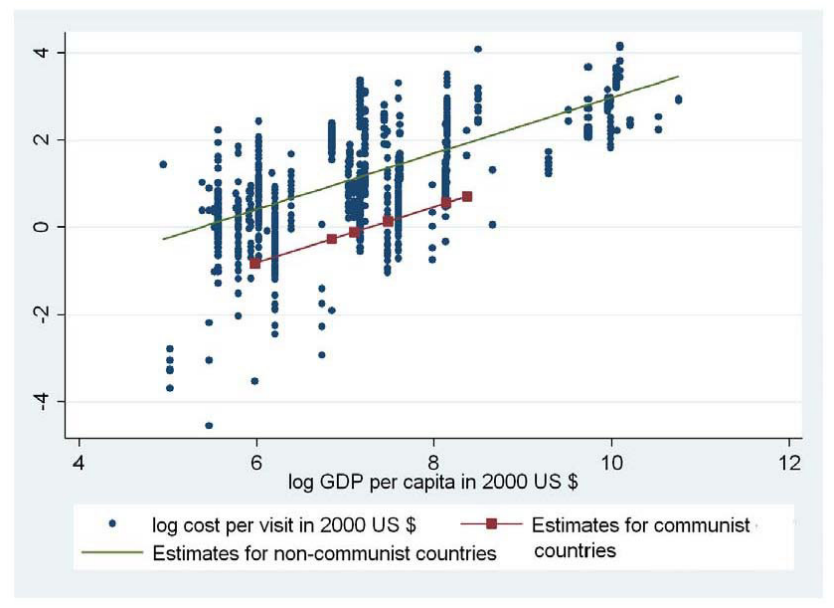
**Predicted values (regression lines) for communist and non-communist countries plotted against the natural log of GDP per capita (X axis).** (Y-axis shows the raw data for cost per visit in natural logs) N = 984.

Figure 1 plots the predicted values from the model against the unit cost data and the level of GDP per capita. The two lines represent the predicted values of the cost per visit (in natural logs), estimated for a public facility with an average capacity use set arbitrarily at 25 visits per provider per day, including capital costs and estimated separately for communist and non-communist countries. The figure confirms that the model has a reasonable fit with the data and illustrates the considerable variability in the observed unit costs within a single country (each column of dots represents a country with a specific GDP per capita).

**Figure 2:**
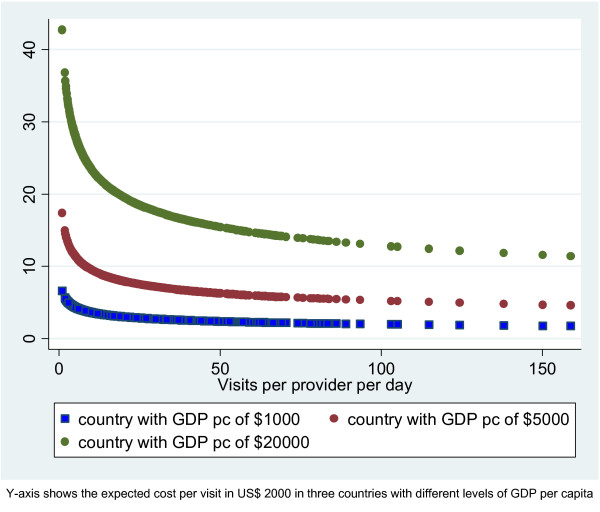
Impact of patient load on unit cost per visit in three settings.

To isolate the impact of the level of capacity utilization on unit costs, we re-estimated the predicted values allowing the capacity level to vary but keeping all other variables constant, including GDP per capita. This is illustrated in Figure 2, which shows the relationship between changes in capacity utilization (x axis) and the level of unit cost per outpatient visit (Y axis), estimated for three settings with different income levels, set at US $1000, $5000 and $20000 for illustration purposes. The figure shows that changes in capacity use can lead to a difference of between $5 and $30 in the estimated costs of an outpatient visit. The estimated costs of scaling up interventions could, therefore, be substantially different depending on the level of capacity utilization that happened to be associated with the data used for the costs of outpatient care.

## Discussion and policy relevance

This paper presents critical evidence on the extent of variability in the cost of a patient visit at primary facilities within and across countries, and the proportion that can be explained by variations in patient load as well as other determinants. While a substantial portion of the observed variability could be explained by the specified determinants, some unexplained variability remained, possibly linked to variables that we could not measure including quality of care, case mix and salary differentials for staff working in remote areas. These variables are likely to explain part of the variability in the observed unit cost data but we did not have the data to explore this.

There are other limitations of this type of analysis that must also be considered when interpreting the results. While the model incorporates a very extensive database on unit costs, much larger than has previously been available, it is always preferable to include more data points. In this case, increasing the number of countries for which observations were available, and having more information on possible explanatory variables, would increase the explanatory power of the model and the validity of the results for extrapolation to a wider number of countries.

We also recognize that the mathematical specification of the model we report here, the log-log form, does not allow the identification of diseconomies of scale if they exist. Cross country studies like this typically use this functional form, which can be interpreted as the downward sloping part of a long-run cost curve. It is possible, as we stated in the introduction, that some countries will face diseconomies of scale if, for example, they have to build new health facilities in isolated areas, and these facilities are not fully utilized. In that case, the higher unit costs of the new facilities can still be estimated from our model – by using the country's observed GDP per capita, for example, and the lower level of capacity utilization associated with the expansion of facilities. Estimating the likely capacity utilization rates associated with the expansion of health facilities to increasingly remote areas is, of course, complex but some experience exists using spatial models to identify the population's physical accessibility to different possible locations of new health facilities [75].

Bearing in mind these limitations, we can still be confident of a number of important conclusions. Firstly, the results show that unit costs are very sensitive to the number of patients seen by providers each day – each 1% increase in patient through-put means, on average, a 27% reduction in the cost per outpatient visit. These variations in capacity utilization can make a difference of up to $30 in the costs per outpatient visit at primary facilities in the same setting, other factors held constant.

This means that estimates of the costs of scaling up, and the resulting estimates of financial needs, that are based on outpatient visit costs taken from a single, or a few studies, could be markedly wrong. These studies could well have capacity utilisation rates that are atypical of the country as a whole. Moreover, they will also be wrong if they do not allow the cost of an outpatient visit to change as coverage increases. Because most of the studies of the costs of scaling up to meet the MDGs do not even report the information on capacity utilization used to derive their outpatient costs estimates, readers can have little confidence that the overall costs that they estimate are even approximately correct.

There are two additional practical uses of the analysis reported in this paper. The first is to apply the model to analyse and adjust locally available unit cost estimates, taking into account differences in capacity use and other determinants. The second is to use the results of the model to estimate the likely unit cost per visit at different levels of capacity use in settings where information on unit costs is not available. There have been several applications of the latter, including estimating the cost-effectiveness of a large set of interventions as part of the WHO-CHOICE [76,77] and the Disease Control Priorities (DCP) projects [78]; and estimating the cost of scaling up health interventions to meet universal coverage of key interventions to address major disease burden such HIV/AIDS [62,79], and interventions to improve maternal and child health [80-82].

Finally, our findings have important implications for the transferability and validity of costing and cost-effectiveness results. General policy decisions should not be based on the results of costing studies that do not report capacity utilization or that base the analysis of the cost of scaling up on current costs of providing care.

## Competing interests

The authors declare that they have no competing interests.

## Authors' contributions

TA constructed the model, performed the analysis and drafted the manuscript. SE and BJ contributed to the selection of variables and model applications. DE participated in the development of the methodology, selection of the model and interpretation of the results. All authors contributed to the writing, and read and approved the final manuscript.
